# Effect of FluoRoquinolones on Aortic Growth, aortic stIffness and wave refLEctionS (FRAGILES study)

**DOI:** 10.3390/life14080992

**Published:** 2024-08-09

**Authors:** Vasiliki Gardikioti, Christos Georgakopoulos, Eirini Solomou, Emilia Lazarou, Konstantinos Fasoulakis, Dimitrios Terentes-Printzios, Konstantinos Tsioufis, Dimitrios Iliopoulos, Charalambos Vlachopoulos

**Affiliations:** 1 First Department of Cardiology, Hippokration General Hospital, Medical School, National and Kapodistrian University of Athens, 11527 Athens, Greece; 2 Department of Urology, Hippokration General Hospital, 11527 Athens, Greece; 3 Laboratory of Experimental Surgery and Surgical Research, Medical School, National and Kapodistrian University of Athens, 11527 Athens, Greece

**Keywords:** quinolones, aortic aneurysm, aortic disease, arterial stiffness, pulse wave velocity, augmentation index

## Abstract

**Background**: The widespread use of fluoroquinolones has been associated with the formation, dissection, and rupture of aortic aneurysms. Arterial biomarkers are established predictors of cardiovascular events. The present study was designed to investigate the effect of quinolones on arterial stiffness and aortic size for the first time. **Methods**: We studied 28 subjects receiving short-term (<15 days) antibiotic therapy involving quinolones and 27 age- and sex-matched subjects receiving an alternative to quinolone antibiotics. The follow-up period was approximately 2 months. The study’s primary endpoint was the carotid–femoral pulse wave velocity (cfPWV) difference between the two groups 2 months after therapy initiation. Secondary endpoints were the augmentation index corrected for heart rate (AIx@75) and sonographically assessed aortic diameters 2 months after the initial treatment. **Results**: Subjects had similar values of arterial biomarkers, blood pressure measurements, and aortic diameters at baseline. At follow-up, no significant change was observed between the two groups regarding the hemodynamic parameters and arterial biomarkers (*p* > 0.05 for all), i.e., cfPWV (7.9 ± 2.6 m/s for the control group vs. 8.1 ± 2.4 m/s for the fluoroquinolones group; *p* = 0.79), AIx@75 (22.6 ± 9.0% for the control group vs. 26.6 ± 8.1% for the fluoroquinolones group; *p* = 0.09), and aortic diameters. **Conclusions**: To our knowledge, FRAGILES is the first study to provide insights into the possible effects of fluoroquinolones on arterial biomarkers, showing that, at least in the short term, treatment with fluoroquinolones does not affect aortic function and diameter.

## 1. Introduction

Aneurysmal dilatation of the aorta is a potentially dangerous condition, the early diagnosis and strict monitoring of which is essential as aortic dissection or rupture is associated with exceptionally high mortality [1,2]. Epidemiological studies report that the annual incidence of aortic aneurysm rupture is 3 to 20 cases per 100,000 people [3,4], which rises to 130 cases per 100,000 individuals among the elderly population [5]. Established risk factors for developing aortic aneurysm to date include age, smoking, hypertension, atherosclerosis, and male sex, as well as congenital arterial wall abnormalities often related to Marfan, Ehlers–Danlos or Loeys–Dietz syndromes [1,2,3,4,5].

Recently, the association of fluoroquinolone use with an increased incidence of aortic aneurysm formation and acute aortic syndrome has led to the issuing of an FDA drug safety announcement advising caution in the use of this class of antimicrobial agents. Despite their excellent utility in the treatment of the most resistant infections, numerous studies have demonstrated the adverse effects of fluoroquinolones on collagen-rich structures other than the aorta [6,7]. Epidemiological studies linking the use of fluoroquinolones with the formation, dissection, and rupture of aortic aneurysms have led to extensive research during the last decade, driven by the widespread use of fluoroquinolones and the criticality of the induced disease [8,9,10,11,12,13,14,15,16,17,18,19,20,21,22,23] (Table S1 in Supplementary S1).

Arterial biomarkers reflect early functional and structural changes and act as surrogate endpoints of arteriosclerosis, which is defined as medial vascular fibrosis and calcification [24]. Carotid–femoral pulse wave velocity (cfPWV) is the gold-standard index for the assessment of aortic stiffness, which has been proven to confer incremental predictive utility for adverse cardiovascular events and all-cause mortality [24,25,26,27,28,29,30]. Along with the augmentation index (AIx), a composite measure of wave reflections and arterial stiffness, these established arterial biomarkers have been proposed to identify the “wear and tear” of the arterial system with age-related, mechanical, and other causes of strain [24,30].

The present study was designed to provide information on the short-term effect of antibiotic treatment on the aortic structure and function of patients on fluoroquinolones compared to alternative antibiotic regimens for 2 months.

## 2. Materials and Methods

### 2.1. Study Design

The effect of FluoRoquinolones on Aortic Growth, aortic stIffness and wave refLEctionS (FRAGILES) study is a case–control clinical study of two groups (fluoroquinolones versus alternative antibiotics, e.g., β-lactams, macrolides, trimethoprim/sulfamethoxazole) receiving short-term (<15 days) antibiotic therapy with quinolones or an alternative to quinolone antibiotic. The study aimed to investigate the effects of quinolones on arterial stiffness, reflected waves, and aortic size to draw conclusions on the cardiovascular safety of quinolone exposure. Designed to reflect current clinical practice, the present study focused on two time points (designated Visit V1 and Visit V2) with a total follow-up period of 2 months after therapy initiation. The study complied with the Declaration of Helsinki and was approved by our Institutional Review Board, and all participants gave written informed consent.

### 2.2. Patient Selection

The study population consisted of adult patients (18 years and older) who had either been diagnosed with an uncomplicated infection (e.g., respiratory or urinary tract infection) or would undergo a planned procedure/surgery and had an indication for antibiotic treatment consisting of a fluoroquinolone or an alternative antibiotic.

#### 2.2.1. Inclusion Criteria

To be eligible for the study, patients had to meet the following criteria:

- Provide written informed consent before enrollment;
- Be over 18 years of age, both sexes;
- Have been diagnosed with an uncomplicated infection (i.e., without signs of severe sepsis), or have been scheduled for a procedure/surgery and thus have an indication to receive a fluoroquinolone or an alternative antibiotic.

#### 2.2.2. Exclusion Criteria

Any of the following was considered a criterion for exclusion from the study:

- Participation in the design and conduct of the study;
- Hypersensitivity to the active substance or any excipients of the antibiotics being administered;
- Long QT syndrome;
- Malignancy, severe autoimmune disease, or any other current illness (e.g., neurological or psychiatric) that could affect or prevent unimpeded participation in the study;
- Severe renal impairment (eGFR <30 mL/min/1.73 m^2^);
- Severe hepatic impairment, defined as liver cirrhosis, elevated transaminases or alkaline phosphatase more than three times their upper limit of the normal value, or hyperbilirubinemia;
- Severe obstructive sleep apnea;
- Pregnant or breastfeeding women or women of childbearing potential not taking contraceptives;
- Need for simultaneous use of more than one antibiotic;
- Life expectancy of less than one year;
- Inability to comply with the study protocol, e.g., due to planned movement away from the study site or due to alcohol or other substance use.

### 2.3. Recruitment and Study Measurements

Recruitment was performed during the first medical interaction with the patient that led to a diagnosis of infection or at the time of admission for a planned procedure, exactly before antibiotic treatment initiation. Potential participants were screened according to inclusion/exclusion criteria. Every patient eligible for inclusion was recruited in the quinolone or the alternative antibiotic therapy group based on the attending physician’s discretion about the type of treatment. The recruitment procedures employed, and a graphic representation of our study timeline are provided in the Supplementary Data (Supplementary S2 and S3, Figure S1).

During Visit V1, demographic characteristics and personal and familial medical history were documented for each participant. Hemodynamic and vascular measurements were performed according to the study protocol in each visit: inclusion Visit V1 (time 0) and Visit V2 (2 months after Visit V1). The same examiners performed all measurements throughout the study and were blinded to the details of each participant. On both occasions, subjects fasted and abstained from smoking, caffeine, ethanol, and flavonoid-containing beverage intake for at least 6 h before measurements. All vascular studies were performed in a quiet, temperature-controlled room at 23 °C. After a 15-min rest period, measurements were conducted in the supine position in a fixed order. A detailed description of the study protocol is provided in Supplementary S4.

#### 2.3.1. Measurement of Peripheral Blood Pressure

Peripheral (brachial) blood pressure (BP) was measured using a validated automated oscillometric device (BP-203RPE III [VP-1000], Omron Colin, Japan). BP measurements were taken using a standardized method (Supplementary S4.1) at the beginning of each session, before any other intervention or examination, in both Visits V1 and V2.

#### 2.3.2. Evaluation of Aortic Stiffness

Aortic stiffness was assessed via cfPWV using a validated noninvasive device (Complior©, Artech Medical, Paris, France). cfPWV equals the distance in meters divided by transit time (TT) in seconds. This distance was calculated by subtracting the distance between the measurement site of the right carotid artery and the sternal notch (carotid–notch) from the distance between the site of the right femoral artery and the sternal notch (femoral–notch). For further details, please refer to Supplementary S4.2.

#### 2.3.3. Pulse Wave Analysis

The aortic augmentation index corrected for heart rate at 75 beats per minute (AIx@75) is a composite, indirect index of wave reflections and aortic stiffness. AIx@75 was calculated non-invasively using a validated, commercially available system (SphygmoCor© CVMS CP, AtCor Medical, Sydney, Australia), which employs the principle of applanation tonometry and a dedicated software to conduct pulse wave analysis (PWA). For a detailed description, please refer to Supplementary S4.3.

#### 2.3.4. Measurement of Aortic Diameters

The examination of each participant included a transthoracic echocardiogram (TTE) at baseline and at the 2-month follow-up. The aorta was measured at four levels—the aortic annulus, sinuses of Valsalva, sinotubular junction, and the ascending aorta at approximately 10 cm from the aortic annulus according to a standardized method (Supplementary S4.4).

Afterward, each participant’s abdominal aortic diameters were thoroughly evaluated ultrasonographically at baseline and at the 2-month follow-up, following a standardized protocol (Supplementary S4.5).

#### 2.3.5. Laboratory Determinations

High-sensitivity C-reactive protein (hsCRP) was measured via immunonephelometry (Dade Behring, Marburg, Germany) at baseline and at the 2-month follow-up.

#### 2.3.6. Follow-Up

All patients were followed up 2 months after initiating the assigned antibiotic therapy. An independent clinical endpoint investigator was responsible for adjudicating any adverse events.

### 2.4. Study Outcomes

#### 2.4.1. Primary Outcome

The study’s primary outcome was the difference in cfPWV between the two treatment groups 2 months after initiating therapy with a quinolone or an alternative to quinolone antibiotic.

#### 2.4.2. Secondary Outcomes

The secondary outcomes of the study included the following:

- The difference in the mean value of the aortic AIx@75 between the two treatment groups at 2 months after the initial administration of quinolone or of an alternative to quinolone antibiotic therapy;
- The difference in the sonographically evaluated aortic diameters at the ascending and abdominal aorta level between the two treatment groups at 2 months after the initial administration of quinolone or an alternative to quinolone antibiotic therapy.

### 2.5. Statistical Plan

#### 2.5.1. Sample Size Calculation

As there is no previous study evaluating the effect of quinolones on aortic stiffness, the sample size calculation was based on the assumption that quinolones would be associated with a minimum clinical change of at least 0.8 m/s of cfPWV in the quinolone treatment group compared with a control group matched in a 1:1 ratio. Therefore, it was estimated that a minimum of 25 patients should participate in each group (50 in total) to provide 80% potency at a significance level of α = 0.05. Due to the study’s relatively short duration, a withdrawal rate of less than 5% was expected; therefore, recruiting at least 54 patients (27 in each group) was considered sufficient to conclude the study’s primary endpoint.

#### 2.5.2. Statistical Analysis

Results for continuous variables are expressed as mean ± standard deviation. Descriptive data for continuous variables are expressed as mean, standard deviation, and percentages for frequencies. Categorical variables are represented with frequency tables (*n*, %). The regularity of the distribution of continuous variables was examined with the Kolmogorov–Smirnov test to determine whether parametric tests could be used to analyze the collected data. Transformations were applied to the variables to resemble the normal distribution when necessary. The difference in continuous variables between the various predetermined time points was assessed in pairs using Student’s *t*-test or the Wilcoxon signed-rank test for data that were not normally distributed. An imputation strategy was employed in the event of missing values at random. Specifically, if a participant had missing data at a particular time point, the missing value was imputed using the participant’s last known value, assuming that the missing values occurred randomly and were not systematically related to the unobserved values. All statistical tests were bilateral, with a 5% level of statistical significance. Statistical analysis, as well as tables and graphs, were created using IBM SPSS^®^ Statistics for Windows, Version 24.0 (IBM Corp., Armonk, NY, USA).

## 3. Results

### 3.1. Baseline Characteristics

Table 1 presents the baseline characteristics of the population (*n* = 55). The group of subjects treated with antibiotics alternative to fluoroquinolones had higher rates of coronary artery disease (CAD) and heart failure (HF) compared to the control group. Apart from these two variables, both groups were well matched (Table 1).

Blood pressure and heart rate were similar between the two groups at baseline (Table 2). Moreover, the baseline inflammatory status, as assessed via hsCRP, was similar between the two groups (Table 2). Interestingly, we observed higher levels of hsCRP in patients with CAD (*p* = 0.001), HF (*p* = 0.001), hyperlipidemia (*p* = 0.032), and renal disease (*p* = 0.042) compared with patients without these conditions or risk factors. Also, the baseline measurements of arterial biomarkers (cfPWV and AIx@75) and aortic diameters were comparable between the two groups (Table 2).

### 3.2. Arterial Biomarkers at 2-Month Follow Up

In our population, 11 patients were lost at follow-up. Consequently, 2 months after Visit 1, only 44 patients were available for re-assessment. Reasons for withdrawal included inability to return due to geographical distance (*n* = 7) and debilitation caused by non-cardiovascular comorbidities (*n* = 4).

**Figure 1:**
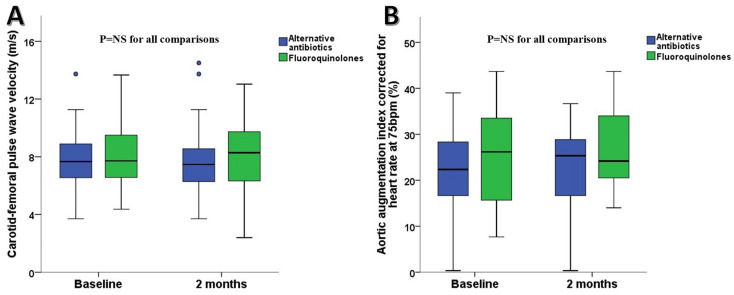
Boxplots of (**A**) carotid–femoral pulse wave velocity levels and (**B**) aortic augmentation index corrected for heart rate between patients on fluoroquinolones and patients on an alternative to fluoroquinolone antibiotics. NS: non-significant.

At the 2-month follow-up, no significant changes in arterial biomarkers were observed in either of the study groups compared to their respective baselines. Additionally, no significant change was observed in arterial biomarkers in the two groups (Table 2, Figure 1). Blood pressure and heart rate were similar between the two groups at 2 months (Table 2). The cfPWV was not different between the two groups at 2 months (7.9 ± 2.6 m/s for the control group vs. 8.1 ± 2.4 m/s for the fluoroquinolones group; *p* = 0.79). Moreover, AIx@75 was not different between the two groups at 2 months (22.6 ± 9.0% for the control group vs. 26.6 ± 8.1% for the fluoroquinolone group; *p* = 0.09) (Figure 1). Even though AIx@75 showed an absolute increase of 1% and 1.2% in the fluoroquinolone and alternative antibiotic regimen groups at the 2-month follow-up, respectively, this change was not statistically significant (Table 2).

### 3.3. Aortic Diameters at 2-Month Follow Up

**Figure 2:**
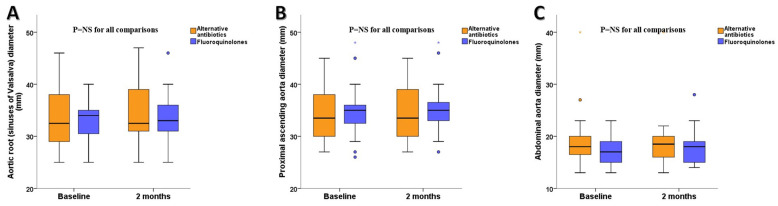
Boxplots of (**A**) aortic root diameters measured at the level of the sinuses of Valsalva, (**B**) proximal ascending aorta diameters, and (**C**) abdominal aorta diameters between patients on fluoroquinolones and patients on an alternative to fluoroquinolones antibiotics. NS: non-significant.

In either group, the aortic diameters did not change significantly from baseline at 2 months (Table 2, Figure 2).

The aortic diameter at the level of the sinuses of Valsalva was not different between the two groups at 2 months (34.1 ± 5.2 mm for the control group vs. 34.9 ± 4.7 mm for the fluoroquinolones group; *p* = 0.96). Moreover, the aortic diameter of the proximal ascending aorta was not different between the two groups at 2 months (34.2 ± 5.2 mm for the control group vs. 35.4 ± 4.7 mm for the fluoroquinolones group; *p* = 0.39). Finally, the maximal diameter of the abdominal aorta was not different between the two groups at 2 months (19.3 ± 6.1 mm for the control group vs. 18.2 ± 3.5 mm for the fluoroquinolones group; *p* = 0.48) (Table 2).

### 3.4. Safety

Participants who received the respective antibiotic regimens did not report any adverse reactions. No significant events were observed.

## 4. Discussion

Our research has demonstrated that short-term treatment (<15 days) with fluoroquinolones or alternative antibiotics does not induce significant changes in aortic stiffness or wave reflections, and this observation was independent of the class of antibiotic used. Furthermore, aortic diameters did not show any notable alteration two months after the administration of fluoroquinolones compared to baseline measurements or alternative antibiotics. Our findings provide significant insights into the impact of short-term fluoroquinolone use on aortic structure and function, making a valuable contribution to the growing body of research in this area.

### 4.1. Aortic Aneurysms and Arterial Biomarkers

The aorta plays a pivotal role in regulating the performance of the left ventricle and arterial function. A comprehensive assessment of the biomechanical properties of the aorta and peripheral arteries should include the evaluation of arterial stiffness and reflected waves [24]. These indices can be measured non-invasively, are readily assessable, and hold significant predictive utility for future events in several disease populations [24,25,26].

Extensive research has been conducted on aortic aneurysms using established arterial biomarkers due to their pathophysiological affinity with the disease. More specifically, aneurysmal dilatation of the aorta has been linked to a significant increase in aortic stiffness [31,32,33,34]. This could be attributed to a decreased volume fraction of elastin and smooth muscle cells, and a concomitant increase in collagen and ground substance in the diseased aortic wall [35]. It is worth noting that patients with abdominal aortic aneurysms exhibit higher levels of carotid artery stiffness compared to those with coronary artery disease, even after adjusting for important risk factors [36], suggesting that the presence of an aneurysm may be a result of generalized arterial wall disease. AIx was also higher in patients with aortic aneurysms (both thoracic and abdominal) as compared to healthy individuals, even though its values have not consistently been associated with the progression rate of aneurysmal dilatation [37]. However, some case–control studies have generated conflicting results, which could be explained by inherent limitations, such as difficulties in matching the patients with controls [38].

### 4.2. Fluoroquinolones and the Risk of Aortic Aneurysm

A plethora of pathogens have been associated with increased cardiovascular risk, as they may elicit pro-atherogenic and pro-arteriosclerotic effects either directly by infecting the vascular wall or indirectly through systemic inflammatory mechanisms [39]. The risk escalates when the infectious agent causes direct injury to the endothelial and myocardial cells [40,41]. Arterial biomarkers have been studied in this context as well, and they have exerted an ability to detect even subtle short-term treatment-related cardiovascular effects [24,42].

The impact of antibiotics on arterial stiffness, on the other hand, has not been adequately investigated [43], even though data published over the last decade suggest a plausible association between fluoroquinolone use and the risk for aortic aneurysm based on pathophysiological mechanisms very similar to those related to arteriosclerosis. As previously noted, fluoroquinolones are known to compromise the integrity of collagen-containing tissues [6,7]. Experimental studies have demonstrated similarities between the aortic wall exposed to quinolones and congenital anomalies of collagen synthesis, such as Ehlers–Danlos syndrome, which involve cystic degeneration of the medial aortic layer and, consequently, an increased risk of aneurysmal dilatation [44]. Further suggested mechanisms include the disruption of the homeostasis of ions essential for collagen production, such as iron, magnesium, and calcium [45], the enhancement of the production and activity of matrix metalloproteinases (MMPs) in the extracellular matrix, and the concomitant hindrance of tissue inhibitors of metalloproteinases (TIMPs). Collectively, these mechanisms may interfere with collagen synthesis and degradation balance, ultimately compromising the integrity of the aortic wall [46,47].

Regarding the clinical manifestations of these molecular processes, one of the seminal studies reporting the association of fluoroquinolones with the development of aortic aneurysms was a large population-based longitudinal cohort study from Canada, which included approximately 1,700,000 individuals [18]. Lee et al. corroborated these findings by studying two large populations, in which the risk of aortic aneurysm or rupture was found to be 2–3 times higher in patients treated with fluoroquinolones compared to matched controls [8,19]. This effect was estimated to last up to 2 months after exposure to the antibiotic. Several individual studies, endorsed by meta-analyses of their results, further support these findings [9,10,11,12,13,14,15,16,17,20,21,22,23,48] (Supplementary S1).

However, more recent data suggest that the purported link between the administration of fluoroquinolones and aortic aneurysm or dissection may not be a genuine causal relationship [49,50,51]. Brown et al., in their analysis of UK primary care records, have attributed any observed association between the two to confounding factors rather than a direct cause-and-effect relationship [50]. The findings of Huh et al. reinforced this notion, concluding that fluoroquinolone administration should not be discouraged by concerns of aortic aneurysm or dissection if clinically warranted [51].

In line with the latter, our results also support the unimpeded use of fluoroquinolones in clinical practice, indicating that the short-term administration of fluoroquinolones or alternative antibiotics does not significantly impact the structure or function of the aorta over two months. Given the mechanisms involved, even subtle fluctuations in arterial biomarkers would be anticipated during this time frame, which is the period during which prior studies document events. It is also worth noting that arterial biomarkers remained stable in patients with an uneventful clinical course. Even though our study was not explicitly designed to evaluate the effectiveness of vascular biomarkers in monitoring fluoroquinolone use, this observation suggests that these biomarkers could potentially be used to monitor patients’ response to antibiotic treatment and may trigger further research in this area.

It is essential to interpret all the above findings prudently, as conflicting findings may compromise therapeutic decisions. Further investigation is warranted, particularly regarding populations at high risk for aortic aneurysm formation or rupture, with whom we should be extremely cautious.

### 4.3. Study Limitations

We acknowledge that despite the meticulous sample size calculation, our modest sample size may limit the generalizability of our findings. An important limitation of our study is the significant number of participant withdrawals, which were nevertheless due to reasons medically irrelevant to quinolone therapy or cardiovascular morbidity. The absence of female participants is another limitation of our study, making it challenging to generalize our findings to the female gender. This issue was also present in the previous research published on the correlation between quinolones and aneurysm rupture risk, in which the percentage of male participants ranged from 45 to 80% (Supplementary S1, Table S1) despite using registries in large-scale epidemiological studies. Importantly, we did not include individuals with a family history of aortic aneurysm and/or aortic dissection, underlying connective tissue disorder, or inherited syndromic thoracic aortic disease. Therefore, although our results are consistent and indicate validity, we should take caution before applying them to populations at high risk of aortic aneurysm formation and/or rupture. All the extensive studies investigating the association between the use of quinolones and aortic aneurysm/rupture also enrolled subjects from the general population. To further examine specific subgroups, such as populations at increased risk of aortic events or subjects on different classes of antibiotics, a broader cohort would be required, but this was out of the primary scope of the study, which was to assess the effect of quinolones on arterial stiffness.

Finally, we did not opt to assess aortic diameters with more precise imaging techniques, such as computed tomography or magnetic resonance imaging, due to logistic reasons and potential risks, such as acute kidney injury and radiation exposure. Instead, we relied on echocardiography, an accurate and reliable imaging tool for diagnosing and monitoring aortic diseases.

## 5. Conclusions

Fluoroquinolones have been associated with aortic dilation, which may exacerbate acute cardiovascular events in individuals with an already distressed aortic wall due to mechanical forces and atherogenic factors. The FRAGILES study, employing established arterial biomarkers, yielded reassuring evidence that short-term use of fluoroquinolones does not impact aortic structure and function over two months. Our findings may aid in risk stratification, clinical decision-making, and follow-up of patients on antibiotic treatment.

## Figures and Tables

**Table 1:** Baseline characteristics of the study participants, based on the type of antibiotic treatment administered.

Variables	FQ (EXP) [*n* = 28]	Antibiotics Alternative to FQ (CTR) [*n* = 27]	*p*-Value
Age (years)	66.1 (10.6)	65.0 (18.4)	0.79
Male gender, *n*	24 (86)	24 (89)	0.72
Weight (kg)	84.8 (12.6)	81.9 (15.7)	0.45
Height (cm)	174.0 (8.5)	174.4 (10.5)	0.87
Waist (cm)	107.3 (10.7)	104.2 (15.7)	0.40
Hip (cm)	107.0 (10.0)	105.2 (16.2)	0.55
BMI (kg/m^2^)	28.0 (3.5)	26.9 (12.5)	0.38
DM, *n*	6 (21)	10 (37)	0.20
HTN, *n*	15 (54)	17 (63)	0.48
Hyperlipidemia, *n*	10 (36)	14 (52)	0.23
Smoking, *n*	11 (39)	7 (26)	0.29
History of CAD, *n*	5 (18)	12 (44)	0.033
HF, *n*	2 (7)	11 (41)	0.003
KD, *n*	2 (7)	3 (11)	0.61

Categorical variables are presented as absolute and relative frequencies. Continuous variables are presented as mean value ± SD for normally distributed variables and the median value (25th–75th percentile) for skewed variables. FQ, fluoroquinolones; EXP, experimental group; CTR, control group; BMI, body mass index; DM, diabetes mellitus; HTN, hypertension; CAD, coronary artery disease; HF, heart failure; KD, kidney disease.

**Table 2:** Hemodynamic, inflammatory, arterial biomarkers, and aortic diameters at Visit V1 and Visit V2, according to the antibiotic treatment group.

Variables	Visit V1			Visit V2		
	FQ (EXP) [*n* = 28]	Antibiotics Alternative to FQ (CTR) [*n* = 27]	*p*-Value	FQ (EXP) [*n* = 28]	Antibiotics Alternative to FQ (CTR) [*n* = 27]	*p*-Value
Brachial SBP (mmHg)	132.9 ± 21.1	128.3 ± 14.3	0.36	130.2 ± 20.6	128.9 ± 15.6	0.79
Brachial DBP (mmHg)	80.9 ± 14.3	77.5 ± 9.3	0.30	79.0 ± 12.9	78.3 ± 11.0	0.85
HR (beats/min)	70.8 ± 13.8	69.6 ± 11.6	0.72	70.8 ± 13.8	69.6 ± 11.6	0.72
AIx@75 bpm (%)	25.6 ± 10.0	21.4 ± 9.9	0.13	26.6 ± 8.1	22.6 ± 9.0	0.09
cfPWV (m/s)	8.1 ± 2.2	7.9 ± 2.1	0.78	8.1 ± 2.4	7.9 ± 2.6	0.79
hsCRP (mg/L)	2.1 (0.9–7.2)	3.1 (1.1–26.9)	0.31	1.5 (0.8–4.0)	2.3 (1.1–18.2)	0.21
Aortic root diameter (sinuses of Valsalva) (mm)	32.7 ± 3.5	33.5 ± 5.3	0.54	34.9 ± 4.7	34.1 ± 5.2	0.96
Proximal ascending aorta diameter (mm)	35.0 ± 5.1	34.0 ± 5.2	0.51	35.4 ± 4.7	34.2 ± 5.2	0.39
Maximal abdominal aorta diameter (mm)	17.2 ± 2.7	19.7 ± 6.6	0.13	18.2 ± 3.5	19.3 ± 6.1	0.48

Continuous variables as mean value ± SD for normally distributed variables and median value (25th–75th percentile) for skewed variables. FQ, fluoroquinolones; CTR, control group; EXP, experimental group; SBP, systolic blood pressure; DBP, diastolic blood pressure; HR, heart rate; AIx@75, augmentation index corrected for heart rate at 75 bpm; cfPWV, carotid–femoral pulse wave velocity; hsCRP, high-sensitivity C-reactive protein.

## Data Availability

The data supporting this study’s findings are available from the corresponding author, C.V., upon reasonable request.

## Supplementary Materials

The following supporting information can be downloaded at: https://www.mdpi.com/article/10.3390/life14080992/s1. Supplementary S1: Table S1. Early studies investigating the association between the use of fluoroquinolones and the formation, dissection, and/or rupture of aortic aneurysms. Supplementary S2. Recruitment. Supplementary S3. Figure S1. Flow diagram of the FRAGILES study. Supplementary S4. Examination protocol. Supplementary S5. Repeatability of vascular measurements.
