# Supplementary material for: Effect of FluoRoquinolones on Aortic Growth, aortic stIffness and wave refLEctionS (FRAGILES study)
Source: Life (Basel). 2024 Aug 9;14(8):992. doi: 10.3390/life14080992 (PMC11355559; doi:10.3390/life14080992)
Supplement: Supplementary file 1 [file life-14-00992-s001.zip › life-3131245-supplementary.pdf]

Effect of FluoRoquinolones on Aortic Growth, aortic stIffness and wave refLEctionS (FRAGILES study)

Supplementary Data

Supplementary S1.

Table S1. Early studies investigating the association between the use of fluoroquinolones and the formation, dissection, and/or rupture of aortic aneurysms.

AUTHOR YEAR OF PUBLICATION, COUNTRY	STUDY POPULATION	AGE (YEARS)	RESEARCH METHOD	PARAMETERS	FOLLOW-UP DURATION	OBJECTIVES/ MAIN OUTCOMES	RESULTS/ CONCLUSION ABOUT ASSOCIATION WITH AA/AD
Lee et al., 2015, Taiwan [8]	1,477 case patients (AA 74% male, AD 71% male) and 147, 700 age and sex- matched control cases (1:100) from the Taiwan National Health Insurance Research Database from 1998 - 2011	AA 74±12 y.o./ AD 66±15 y.o./ controls 71±14 y.o.	Propensity-matched population-based nested case control	First diagnosis of AA/ AD requiring hospitalization.	Current use group, 60 days; past use group, 61 and 365 days; prior-year use group during the prior 1-year period	Risk of developing AA or AD.	Current use of FQ (in the last 60 days) was associated with increased risk for AA or AD (RR, 2.43; 95% CI, 1.83-3.22), and this association was attenuated for past use (between 2 and 12 mo prior) (RR, 1.48; 95% CI, 1.18-1.86).
Daneman et al., 2015, Canada [42]	1,744,360 pts (49% male) from Ontario Registered Persons Database 1997-2012, of whom 657 950 (38%) received at least one FQ during FU	>65 years old	Population-based longitudinal cohort study	AA, AD	30 days	AA; tendon rupture; retinal detachment; death	FQ may contribute to AA (HR 2.72, 95% CI 2.53 to 2.93; adjusted HR 2.24, 95% CI 2.02 to 2.49).
Lee et al., 2018, Taiwan [43]	1,213 hospitalized AA/AD pts (72% male) from the Taiwan National Health Insurance Research Database from 2001 - 2011	71±14 y.o.	Case-crossover study	First diagnosis of AA/ AD requiring hospitalization.	60 days	Comparison of FQ exposure for the same pt across a 60-day period before the AA/AD event (hazard period) and 1 randomly selected 60-day period (reference period) between 60 to 180 days before the AA/AD events.	The use of FQ within 60 days was associated with the highest risk of AA/AD, even after adjustment for infections and co-medications (OR: 2.05; 95% CI: 1.13 to 3.71), especially after prolonged exposure to FQ (OR: 2.41 for 3- to 14-day exposure; OR: 2.83 for >14-day exposure).

Pasternak et al., 2018, Sweden [44]	360,088 FQ treatment episodes (45% male) and equal amount of propensity score matched comparator episodes of amoxicillin use (1:1) from linked nationwide data from Swedish registers 2006-2013	FQ 68±11 y.o./ Amoxicillin 68±10 y.o.	Propensity-matched population-based cohort study	AA, AD	60 days	Association of FQ use with an increased risk of AA/ AD.	The HR for the association with FQ treatment was 1.90 (1.22 to 2.96) for AA and 0.93 (0.38 to 2.29) for AD.
Maumus - Robert et al., 2019, France [45]	5,946 pts with aortoiliac aneurysm or dissection (64% male) from the French Health Insurance Nationwide Databases from 2010-2015	median age 70 years; interquartile range: 62 to 80 years	Case-time control study	Aortoiliac aneurysm or dissection	180 days	Association of FQ use with increased short-term risk of aortoiliac aneurysm or dissection.	There is an increased risk of aortoiliac aneurysms or dissections 30 days after FQ treatment (OR 2.44, 95% CI: 1.31 to 4.57).
Meng et al., 2019, China [46]	3,721 adverse event reports of AA/AD from the US Food and Drug Administration Adverse Event Reporting System 2004-2016	-	Pharmacovigilance study	AA, AD	(timeframe for events after FQ treatment is not specified)	To assess AA/ AD induced by FQ.	FQ treatment is associated with AA/AD, with the risk for AA being higher.
Gopalakrishnan et al., 2020, United States [47]	279,554 pts treated for pneumonia or urinary tract infection with FQ or alternative antibiotic in 2 pairwise 1:1 propensity score–matched cohorts taken from the United States commercial health insurance claims database from 2003-2015	Pneumonia-cohort 64±11 y.o./ Urinary tract cohort 62±10 y.o.	Propensity – matched population-based cohort study	Hospitalization for AA/AD occurring within 60 days following FQ treatment initiation.	60 days	To assess the association of FQ with risk of AA/AD.	There was an increased RR of AA/AD associated with FQ within the pneumonia cohort (HR, 2.57; 95% CI, 1.36-4.86; incidence, 0.03% for FQ vs 0.01% for azithromycin) but not within the urinary tract infection cohort (HR, 0.99; 95% CI, 0.62-1.57; incidence, <0.01% in both urinary tract infection groups).

Sommet et al., 2020, France [48]	172,588 pts treated with FQ and 40,658 with amoxicillin from the Vigibase, WHO Global Individual Case Safety Reports database from 1972 - 2017	-	Pharmacovigi lance study	AA, AD	(timeframe for events after FQ treatment is not specified)	To assess the comparative risk of AA/AD associated with FQ vs amoxicillin.	Treatment with FQ was associated with a higher risk of reporting AA/AD compared to amoxicillin exposure (OR 2.13, 95% CI 1.03–4.37).
Dong et al., 2020, Taiwan [49]	28,948 cases and 289,480 matched controls (1:10) (71.37% male) from the Taiwan population-based health insurance claims database from 2009-2015	67±15 y.o.	Population-based nested case control	First diagnosis of AA and AD requiring hospitalizati on.	60 days	To assess the comparative risk of AA/AD associated with FQ vs other antibiotics with similar indication.	FQ were not associated with an increased AA/AD risk when compared to alternative antibiotics (vs penicillins OR, 1.01; 95% CI, 0.82-1.24 / vs cephalosporins OR, 0.88; 95% CI, 0.70-1.11), when accounting for the underlying infections.

Pt, patient; AA, aortic aneurysm; AD, aortic dissection; FQ, fluoroquinolone; FU, follow up; RR, relative risk; HR, hazard ratio; OR, odds ratio; CI, confidence interval; y.o., years old; mo, months

Supplementary S2.

Recruitment.

The potential participants were identified through a systematic screening process at the 1st Department of Cardiology and also through collaboration with other departments at the Hippokration Hospital of Athens, specifically:

- the Emergency Department, i.e., patients examined by our colleagues for symptoms suggestive of cardiac disease but were rather diagnosed with an uncomplicated infection requiring antibiotic treatment.
- the Urology Department, i.e., individuals who would undergo a planned procedure/surgery and had an indication for antibiotic treatment consisting of a fluoroquinolone or an alternative antibiotic.

Supplementary S3.

Figure S1. Flow diagram of the FRAGILES study.

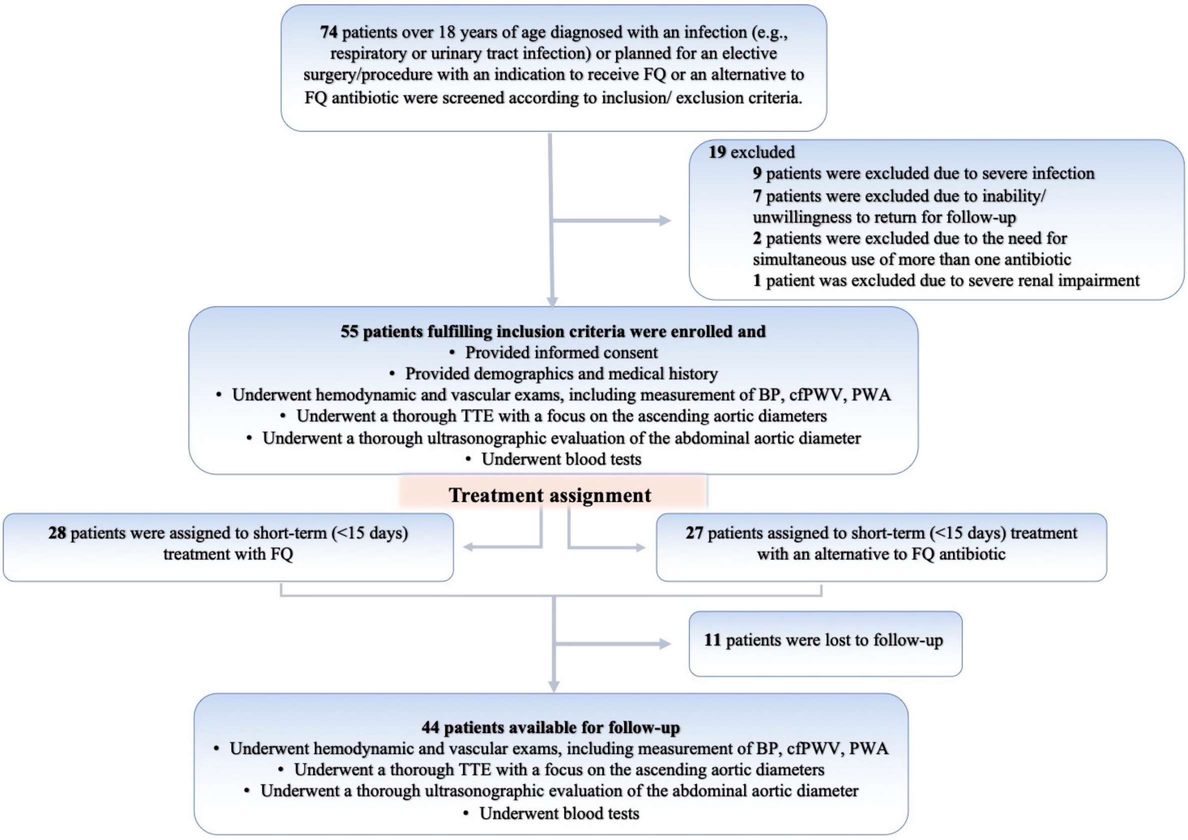

FQ, fluoroquinolones; BP, blood pressure; cfPWV, carotid-femoral pulse wave velocity; PWA, pulse wave analysis; TTE, transthoracic echocardiogram

Supplementary S4.

Examination protocol.

In our dedicated peripheral vessels laboratory at the Hippokration Hospital, we use the validated noninvasive device Complior®, Artech Medical, Paris, France, to measure cfPWV, and the SphygmoCor® CVMS CP, AtCor Medical, Sydney, Australia, for PWA, including the measurement of Augmentation Index (AIx).

The steps of the examination procedure involved in the exact order:

1. The patient, who should have abstained from smoking, consumption of caffeine, food, or drugs, and exercise for at least 6 hours, was laid in the supine position for a 15-minute rest period with the legs uncrossed and the whole body supported correctly.
2. The brachial BP was measured with the standardized method described earlier using the BP-203RPE device (Omron Healthcare Co, Ltd, Kyoto, Japan).
3. The cfPWV measurement was conducted using Complior®, Artech Medical, Paris, France.
4. PWA was conducted by radial applanation tonometry using SphygmoCor® CVMS CP, AtCor Medical, Sydney, Australia.
5. A thorough transthoracic echocardiographic assessment was performed with a focus on the ascending aortic diameters.
6. A thorough ultrasonographic evaluation of the abdominal aorta diameter was conducted.
7. A sample of blood was taken to conduct a series of tests, which included high-sensitivity C-reactive protein.

The order and timing of measurements during each visit were standardized to ensure consistency across all study participants.

S4.1. Peripheral blood pressure measurement

The measurement of peripheral (brachial) blood pressure (BP) was a significant part of our research. Therefore, we ensured that measurements were conducted using a standardized and validated methodology. This standardization involved the patient's position, the device used, the measurement schedule, and the interpretation of results.

In accordance with these guidelines, we employed a validated automated oscillometric device (BP-203RPE III [VP-1000], Omron Colin, Japan) to measure BP. The device instructions were followed to select an appropriate cuff size for each individual based on their arm circumference. Measurements were conducted in a quiet room with a comfortable temperature of 23°C, and patients were instructed to abstain from smoking, caffeine, food, or drugs, and exercise for at least 6 hours before measurement. Patients were placed in a supine position, and the validated automated electronic upper-arm cuff device was placed on the bare arm resting on the individual's side at heart level. The patient's legs were uncrossed, and the whole body was correctly supported. After a 15-minute rest period, the measurements were conducted in the presence of an investigator, without any talking during or between them.

During the initial office visit, BP was measured in both arms, and any possible interarm systolic BP difference >10 mmHg was confirmed with repeated measurements. If confirmed, the arm with the higher BP was used for all subsequent measurements. The final BP value was recorded as the average of two consecutive readings, taken automatically at intervals of 1-2 minutes. BP measurements were taken using the standardized method described above at the beginning of each session, before any other intervention or examination, in both visits, that is, the inclusion visit V1 (time 0) and the follow-up visit V2 (2 months after visit V1).

S4.2. Carotid-femoral pulse wave velocity measurement

The technique of applanation tonometry, utilizing validated tonometers, was employed to acquire carotid arterial waveforms from the region of the neck over the carotid bulb. The path length was calculated by subtracting the distance between the measurement site of the right carotid artery and the sternal notch (carotid-notch) from the distance between the site of the right femoral artery and the sternal notch (femoral-notch), measured directly using a tape measure.

To determine carotid femoral PWV, simultaneous tonometry of the right carotid and right femoral arteries was performed to obtain the respective waveforms, and the pulse transit time was determined using the intersecting tangent algorithm. Each recording consisted of a minimum of 10 cardiac cycles and was considered satisfactory if the standard deviation (SD) for the PWV for that sequence was less than 0.5 m/s. To ensure accuracy, each participant was measured at least twice. If there was a difference of more than 0.5 m/s between the first two measurements, a third measurement was obtained. All recordings were saved separately for each participant and then averaged for statistical analysis. This method aimed to enhance the reliability and accuracy of the measurements.

S4.3. Pulse wave analysis

For pulse wave analysis (PWA), we utilized the SphygmoCor® system, specifically the CVMS CP model, which includes PWA software only.

During a PWA measurement, the peripheral pulse was sampled at the right radial artery of each participant, with the validated SphygmoCor tonometer. Each SphygmoCor measurement involved a 10-second recording of the radial arterial pressure wave. The ascending aortic pressure wave was then determined using specialized software. From the given information, a number of significant variables were determined, including the aortic augmentation index (AIx). We made sure to take at least two measurements from every participant. If we found a difference of more than 5% between the first two measurements, we obtained a third one. We saved all the recordings for each participant separately and then averaged them out for statistical analysis. This approach aimed to enhance the reliability and accuracy of our measurements.

S4.4. Echocardiographic assessment of the aortic root and ascending aorta

For the sonographic assessment of the aorta at various levels, we utilized the General Electric VividTM E90 Ultra Edition device. The transducer used for the visualization of the ascending aorta was GE HealthCare M5Sc-D XDclear Matrix Phased Array Probe, which offers good image quality and resolution for the specific measurements conducted.

The aortic root and ascending aorta were measured in a standardized way. Measurements were obtained at end-diastole (determined with the aid of an implemented rhythm ECG lead) in the parasternal long-axis view, with the transducer set an intercostal space higher than in the typical placement to allow for a focused image of a longer part of the ascending aorta. More specifically, the transducer was placed in the left second or third intercostal space near the sternum of each individual, who was set in the left lateral decubitus position. The aorta was measured at four levels - the aortic annulus, sinuses of Valsalva, sinotubular junction, and the ascending aorta at approximately 10 cm from the aortic annulus. The aortic annulus was measured as an inner diameter, while the other sites of the ascending aorta were measured from leading edge to leading edge as per consensus. In individuals in whom the visualization of the ascending aorta was not optimal from the parasternal view, we also used the suprasternal view to visualize the ascending aorta in a more complete fashion, as this view allows for a better assessment of the distal ascending aorta and the aortic arch. All measurements are taken perpendicularly to the aorta in 2D imaging mode. Real-time zoom was also used to achieve an optimal assessment of the aortic root.

S4.5. Ultrasonographic assessment of the abdominal aorta

A curvilinear probe (GE HealthCare C1-6-D Convex Array Ultrasound Probe with XDclear Transducer Technology) was used to scan each participant's abdominal aorta following a standardized protocol. Participants had abstained from the consumption of food for at least 6 hours to allow for an unobstructed visualization of the abdominal aorta. Each individual was examined in the supine position. First, with the probe on the short axis, the entire length of the abdominal aorta was swept from the diaphragm to the bifurcation of the common iliac vessels in the 2D imaging mode. Measurements were obtained at multiple sites, e.g., at the level of the diaphragm, the upper mesenteric arteries, the renal arteries, and at the bifurcation, with both anteroposterior and transverse measurements from leading edge to leading edge, to identify maximal diameters. The same procedure was followed on the long axis, measuring the anteroposterior diameter. In our comparisons between visit 1 and visit 2 of each individual, we used the maximum diameter measured at each site of the abdominal aorta. In our final statistical model, we utilized the maximal diameter of all the abdominal aorta diameters measured in each individual, which, in nearly all cases, was the infra-diaphragm one.

Supplementary S5

Repeatability of vascular measurements

Repeatability in our laboratory for determining cfPWV and augmentation index (AIx) has been previously defined according to the Bland-Altman method. The repeatability coefficient was calculated as defined by the British Standard Institution, i.e., according to the following formula: repeatability coefficient = $2 \times \sqrt{(\Sigma di2/N)}$, where N is the sample size and di, the difference between the two measurements in a pair. The repeatability coefficient values were 0.57 m/sec and 6% for cfPWV and AIx, respectively. Furthermore, our intraclass correlation of coefficient for all measurements is greater than 0.9, providing very good reliability.
